# Role and regulation of FOXO3a: new insights into breast cancer therapy

**DOI:** 10.3389/fphar.2024.1346745

**Published:** 2024-03-04

**Authors:** Wenqiu Mei, Bingyin Mei, Jing Chang, Yifei Liu, Yanhong Zhou, Ni Zhu, Meichun Hu

**Affiliations:** ^1^ Key Laboratory of Environmental Related Diseases and One Health, School of Basic Medical Sciences, Xianning Medical College, Hubei University of Science and Technology, Xianning, China; ^2^ Department of Neurology, Ezhou Central Hospital, Ezhou, China; ^3^ School of Biomedical Engineering, Xianning Medical College, Hubei University of Science and Technology, Xianning, China; ^4^ Department of Medical School of Facial Features, Xianning Medical College, Hubei University of Science and Technology, Xianning, China

**Keywords:** FoxO3a, breast cancer, cell cycle, EMT, apoptosis, drug resistance, ncRNA, small molecular drugs

## Abstract

Breast cancer is the most common malignancy in the world, particularly affecting female cancer patients. Enhancing the therapeutic strategies for breast cancer necessitates identifying molecular drug targets that effectively eliminate tumor cells. One of these prominent targets is the forkhead and O3a class (FOXO3a), a member of the forkhead transcription factor subfamily. FOXO3a plays a pivotal role in various cellular processes, including apoptosis, proliferation, cell cycle regulation, and drug resistance. It acts as a tumor suppressor in multiple cancer types, although its specific role in cancer remains unclear. Moreover, FOXO3a shows promise as a potential marker for tumor diagnosis and prognosis in breast cancer patients. In addition, it is actively influenced by common anti-breast cancer drugs like paclitaxel, simvastatin, and gefitinib. In breast cancer, the regulation of FOXO3a involves intricate networks, encompassing post-translational modification post-translational regulation by non-coding RNA (ncRNA) and protein-protein interaction. The specific mechanism of FOXO3a in breast cancer urgently requires further investigation. This review aims to systematically elucidate the role of FOXO3a in breast cancer. Additionally, it reviews the interaction of FOXO3a and its upstream and downstream signaling pathway-related molecules to uncover potential therapeutic drugs and related regulatory factors for breast cancer treatment by regulating FOXO3a.

## 1 Introduction

Cancer is a prevalent malignant disease worldwide and ranks second in terms of mortality, following cardiovascular disease (Liu et al., 2022a). Over the past years, the incidence and mortality of cancer have been rapidly increasing due to the aging population growth (Yan et al., 2020; Mendez-Couz et al., 2022). GLOBOCAN 2020 data, surveys that breast cancer has overtaken lung cancer as the most common cancer globally (Sung et al., 2021; Wu et al., 2022). Despite extensive laboratory, epidemiological and clinical research conducted over several decades, the occurrence of breast cancer remains on the rise (Britt et al., 2020). Different molecular subtypes of breast cancer have specific targeted drugs that can effectively improve short-term prognosis. However, patients with triple-negative breast cancer (TNBC) often have a poor prognosis due to the high tumor heterogeneity. Endocrine therapy is the most effective treatment for estrogen receptor (ER)-positive early breast cancer. Classical endocrine therapy drugs include Tamoxifen (TAM), steroidal (exemestane), and non-steroidal (letrozole or anastrozole) aromatase inhibitors. Chemotherapy is the primary treatment for patients diagnosed with TNBC. Anthracycline and paclitaxel chemotherapy drugs are commonly used for TNBC patients in case of the failure of endocrine therapy. (Poggio et al., 2018). Trastuzumab and pertuzumab are the representative drugs for treating HER2-positive breast cancer (Valabrega et al., 2007; Swain et al., 2015). These therapies have efficacy to prolong the survival time of breast cancer patients and reduce mortality rates (Jiawei et al., 2022). Despite the overall survival benefits of endocrine therapy for ER-positive breast cancer patients, around one-third of them will eventually develop drug resistance (Rock et al., 1990). Although surgery combined with chemotherapy can extend the overall survival of TNBC patients, those patients with remaining lesions have a higher risk of recurrence. TNBC exhibits significant heterogeneity within tumors and often develops resistance to chemotherapy drugs (Symmans et al., 2017). Consequently, conducting in-depth research will greatly contribute to the development of personalized diagnosis and treatment approaches for breast cancer patients (Yan et al., 2020).

FOXO3a, also known as FOXO3, is a number of the FOXO subfamily of forkhead transcription factors. It is found in a multitude of tissues and organs and plays a significant role in numerous biological processes (Benayoun et al., 2011; Kakuki et al., 2022). FOXO3 has extensive connection with some autoimmune diseases (Xu et al., 2021). In cancer, FOXO3a dysfunction was found to be closely related to tumor drug resistance, metastasis, and apoptosis. The role of FOXO3a in breast cancer development has attracted intensive concerns of numerous studies and become increasingly evident (Liu et al., 2022b). Therefore, FOXO3a targeted therapy will provide a new solution for the treatment and prevention of breast cancer. This review focuses on summarizing the possible role of FOXO3a in breast cancer. Additionally, we provide an overview of the potential targets in the upstream and downstream signaling pathways of FOXO3a and the latest progress in therapeutic strategies.

## 2 Structure of FOXO3a

**FIGURE 1:**
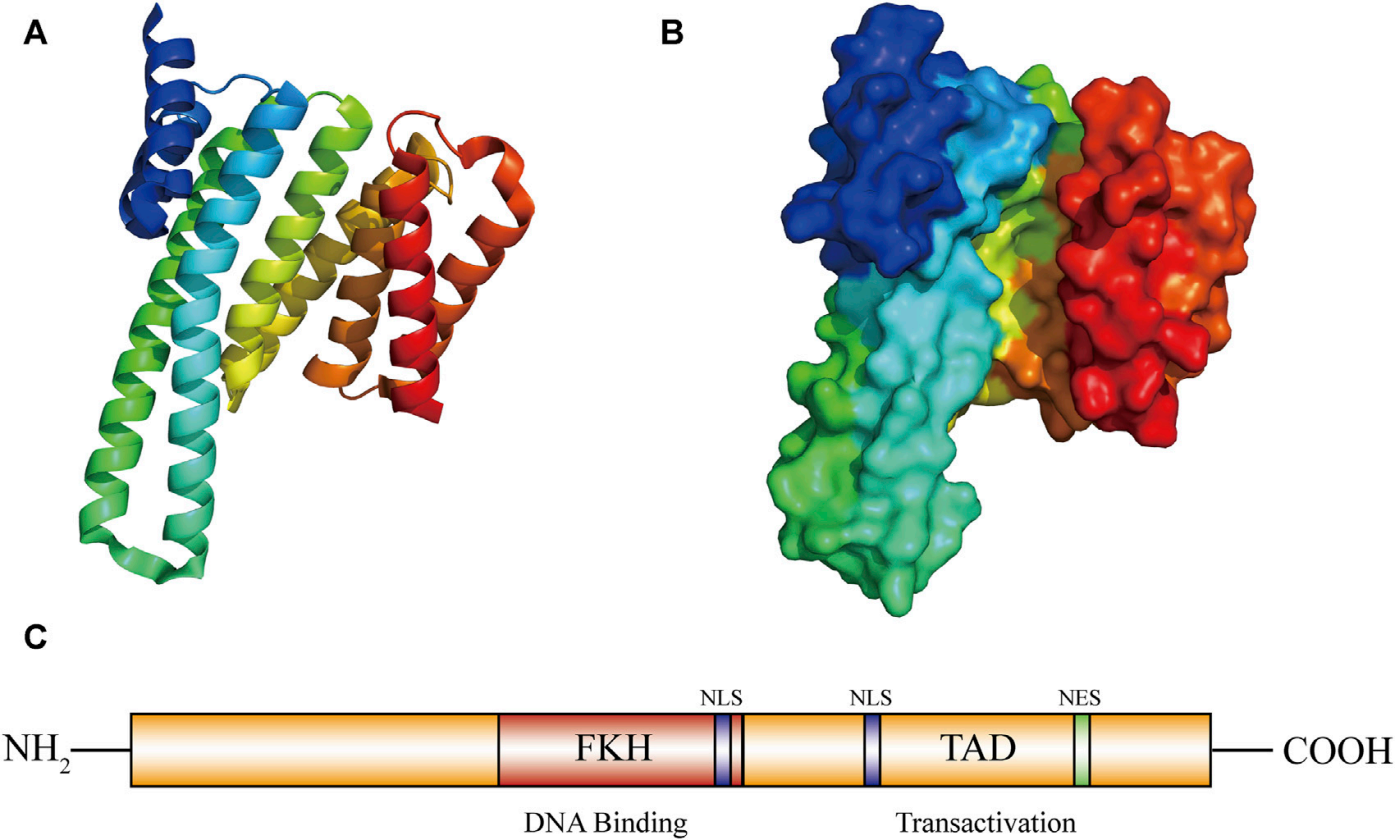
Graph for FOXO3a protein structure. Structure of FOXO3a. **(A,B)** FOXO3a protein 3D structure diagram. **(C)** FOXO3a contains five domains: 1 forkhead winged helix-turn-helix DNA binding domain (FKH); 2 nuclear localization sequence domians (NLS); 1 C-transactivation domain (TAD-C); 1 nuclear export sequence domian (NES).

The FOXO3a gene is located on chromosome 6q21, contains two introns and three exons, and encodes a functional FOXO3a protein of about 71 kDa (Donlon et al., 2017). The FOXO3a protein contains five domains, including a forkhead wing-turn-helix DNA domain (FKH), two nuclear localization sequences (NLS), a nuclear export sequence (NES) and a C-terminal transactivation domain (TAD) (Liu et al., 2018) (Figure 1). The C-terminus serves as a nuclear localization signal, contains a protein kinase B (PKB/AKT) phosphorylation site and a 14-3-3 binding motif, and plays a key role in the stability of the FOXO3a protein and DNA complex (Xu et al., 2021). The interaction between FOXO3a and other proteins, like estrogen receptor alpha and sirtuin 1 (SIRT1), can be facilitated by these domains (Liu et al., 2022b). These domains are also closely related to the function of FOXO3a′s transcriptional activity. FOXO3a can directly bind to the homologous FHRE site, thereby regulating the expression of its target genes. This regulation further influences cell cycle arrest, apoptosis, metabolism, differentiation and oxidative stress (Amente et al., 2011).

## 3 Function of FOXO3a

FOXO3a is a crucial factor in various autoimmune diseases such as systemic lupus erythematosus (SLE), rheumatoid arthritis (RA), inflammatory bowel disease (IBD), ankylosing spondylitis and others. FOXO3a has the ability to govern immune tolerance and homeostasis through its regulation of T cells, B cells, dendritic cells and other immune cells (Xu et al., 2021). In heart disease, FOXO3a can reverse cardiac hypertrophy (Tseng et al., 2013) and attenuate myocardial ischemic injury (Guan et al., 2016). FOXO3a has also been shown to be a pivotal transcription factor in the signaling pathway accountable for congenital abnormalities caused by gestational diabetes (Wang et al., 2015). There is also research suggesting that FOXO3a may be beneficial for high glucose exposure and diabetes (Maiese et al., 2008). In the realm of scientific investigations, substantial datasets in recent years have provided compelling evidence supporting the significance of FOXO3a in the development of multiple biological events, such as cancer.

## 4 The role of FOXO3a in cancer

Numerous studies have consistently demonstrated that the expression of FOXO3a is impaired in cancer. Meanwhile, FOXO3a is subject to the crosstalk with multiple other signaling pathways. The post-translational modification (PTM) of FOXO3a protein and FOXO3a′s regulation by ncRNA have also been found to have a significant association with the malignancy, occurrence, and progression of cancer.

### 4.1 PTM regulates FOXO3a in cancer

PTM of proteins refers to the chemical modifications that occur after the protein is translated from the mRNA. These modifications, such as phosphorylation, acetylation, and ubiquitination, play crucial roles in various cancers. They are involved in processes like cell proliferation, apoptosis, DNA damage and cell drug resistance (Liu et al., 2018; Liu et al., 2022b). FOXO3a, for instance, undergoes phosphorylation by upstream kinases such as AKT, extracellular signal-regulated kinase (ERK), serum/glucocorticoid kinase (SGK), and IκB kinase β (IKKβ). AKT is capable of phosphorylating three specific serine/threonine residues (Thr32, Ser253 and Ser315) of FOXO3a (Brunet et al., 1999). When FOXO3a is phosphorylated, it relocates from the nucleus to the cytoplasm and interacts with 14-3-3. Subsequently, FOXO3a is degraded by the ubiquitin-proteasome pathway, thereby facilitating the initiation and progression of cancer (Bader et al., 2005).

### 4.2 Crosstalk between FOXO3a and other signaling pathways

Multiple studies have demonstrated the involvement of FOXO3a in various signaling pathways in cancer. The downregulation of FOXO3a has been shown to activate AKT. This activation of the AKT/FOXO3a pathway promotes cell proliferation. It is observed that the downregulation of FOXO3a promotes radioresistance in nasopharyngeal cancer by inducing epithelial-mesenchymal transition (EMT) and Wnt/β signaling pathways (Lin et al., 2019). Yunqi Zhu et al. found that WT1 interacting protein (WTIP) enhanced the expression of p53 upregulated modulator of apoptosis (PUMA) by upregulating FOXO3a and facilitating its nuclear translocation, thereby increasing apoptosis in acute myeloid leukemia cells (Zhu et al., 2021). Similarly, Zhiwei Zhao discovered that FOXO3a, in collaboration with c-Jun, upregulates the expression of PUMA to promote apoptosis of cisplatin-resistant ovarian cancer cells (Zhao et al., 2012). Another study by Hao Liu et al. revealed that FOXO3a functions as a negative regulator of EMT in prostate cancer. It inhibits the expression of β-catenin through the activation of miRNA34b/c. Additionally, FOXO3a can directly bind to β-catenin and inhibit its transcriptional activity (Liu et al., 2015).

### 4.3 ncRNA regulates FOXO3a in cancer

In recent years, several non-coding RNAs (ncRNAs) including long non-coding RNAs (lncRNAs), circular RNAs (circRNAs) and microRNAs (miRNAs) have been identified as factors involved in the pathological progression of cancer. Moreover, ncRNA has become an increasingly important therapeutic target in cancer, and numerous studies demonstrating the involvement of FOXO3a in the regulatory network by ncRNA. Specifically, FOXO3a has been identified as a target of miRNA in different types of cancers. Cao et al. found that miR-182-5p inhibited the expression of FOXO3a by binding to sites 72–79 in the FOXO3a 3′UTR. Overexpression of miR-182-5p induces G1 arrest by inhibiting AKT/FOXO3a signaling in renal cell cancer (Ni et al., 2014). In addition, downregulation of FOXO3a can activate AKT, and miR-182-5p upregulates Bcl-2 and Bcl-xl, the two downstream apoptosis inhibitors of AKT, thereby promoting cell proliferation and activating the AKT/FOXO3a pathway (Xu et al., 2014; Cao et al., 2018). In prostate cancer, miR-223-3p may increase the chemosensitivity of prostate cancer cells while reducing the radiosensitivity to its target FOXO3a. MicroRNAs such as miR-592, miR-590-3p and miR-96 can also regulate FOXO3a to regulate cell proliferation and tumorigenesis (Habrowska-Gorczynska et al., 2021). Additionally, CircMRPS35 increases the acetylation of H4K5 in the promoter region of FOXO1 and FOXO3a by recruiting KAT7, thereby upregulating the upregulation of FOXO1 and FOXO3a, and finally inhibiting the proliferation and invasion of gastric cancer cells (Jie et al., 2020). In summary, the expression of FOXO3a in cancer can be regulated by multiple ncRNAs, but the specific mechanism awaits further investigation.

## 5 The role of FOXO3a in breast cancer

As the molecular mechanism of FOXO3a in various malignant tumors has been revealed, recent studies have increasingly focused on the therapeutic potential of FOXO3a in breast cancer intervention. Investigating the action and mechanism of FOXO3a on different phenotypes in breast cancer has emerged as a prominent research topic. Activation of FOXO3a through various pathways not only promotes apoptosis and induces cell cycle arrest, but also plays a suppressing role in EMT and drug resistance in breast cancer cells. It is important to note that FOXO3a does not act independently in breast cancer phenotypes, but rather exhibits interconnections among different phenotypes (Liu et al., 2022b). Overexpression of FOXO3a and its nuclear translocation has been found to inhibit the development of breast cancer through these phenotypes. Based on existing studies, this article provides a comprehensive summary of the role of FOXO3a in these phenotypes.

### 5.1 FOXO3a and apoptosis in breast cancer

Evasion of apoptosis is one of the major steps in tumorigenesis and progression (Wang et al., 2018; Hanahan, 2022). Apoptosis has both intrinsic and extrinsic pathways, and FOXO3a exerts both important roles in breast cancer cell apoptosis.

#### 5.1.1 FOXO3a and intrinsic apoptosis in breast cancer

Intrinsic apoptosis mainly acts through mitochondria and is heavily regulated by the Bcl-2 protein family. This family consists of antiapoptotic proteins such as Bcl-2, (Bcl-xl) and myeloid cell leukemia 1 (Mcl-1), as well as proapoptotic proteins. These pro-apoptotic proteins can be further categorized into the BH123 protein group: Bcl-2 associated X (Bax) and Bcl-2 antagonist killer (Bak), and the apoptosis-initiating protein group: Bcl-xl/Bcl-2 associated death promoter (Bad), Bcl-2 homology 3 interacting domain death agonist (Bid), Bcl-2 interacting mediator of cell death (Bim), PUMA, *etc.*, which contain the Bcl-2 homology 3 interacting domain (BH3) (Xiong et al., 2014). BH3 domain-containing members of the Bcl-2 family undergo oligomerization and incorporation into the membrane of mitochondria. This leads to alterations in the permeability of the mitochondrial membrane, resulting in the loss of transmembrane potential and the subsequent liberation of cytochrome C (Harrington et al., 2023). Subsequently, cytochrome C associates with apoptotic protease activating factor-1 (Apaf-1) and Pro-caspase-9, leading to the activation of Caspase-9, which in turn reactivates Caspase-3 and 7. This cascades further facilitates the transmission of the apoptotic signal downstream (Chan et al., 2006). Bim, through its ability to hinder the activity of anti-apoptotic proteins and its involvement in the downstream executioner Bax and Bak, exhibits the capability to trigger apoptosis. FOXO3a is found in MCF-7 breast cancer cells, functions as a transcription factor for Bim and facilitates the process of apoptosis in this context (Sunters et al., 2003). The study conducted by Stan et al. found that FOXO3a and Bim were responsible for the induction of Withaferin A (WA) in MCF-7 and MDA-MB-231 cells, and WA’s pro-apoptotic effects through triggering breast cancer cells’ intrinsic apoptosis was attributed to the involvement of FOXO3a and Bim (Stan et al., 2008). In TNBC, PUMA is upregulated by BEZ235 (an mTOR inhibitor), through the facilitation of FOXO3a translocation from the cytoplasm to the nucleus. Additionally, MCL-1 is downregulated at the translational level. The alterations in MCL-1 and PUMA trigger the liberation of apoptosis regulators Bim, Bax and Bak, thereby initiating the activation of the mitochondria-mediated intrinsic apoptosis pathway (Li et al., 2018). Eustace et al., discovered that the upregulation of FOXO3a expression led to a decrease in cellular flice-like inhibitory protein (c-FLIP) expression. Consequently, downregulation of c-FLIP promoted the increase of Caspase-8 activation, which may lead to the cleavage of Bid to induce the mitochondria-mediated intrinsic apoptotic pathway and increase the activation of caspase-9, thus promoting breast cancer SKBR3-L cells intrinsic apoptosis (Eustace et al., 2018).

#### 5.1.2 FOXO3a and extrinsic apoptosis in breast cancer

The extrinsic pathway of apoptosis is initiated by the binding of Fas ligand (FasL) or tumor necrosis factor-α (TNF-α) to their respective receptors. This activation of death receptors on the cell surface ultimately leads to apoptosis. Fas, which belongs to the tumor necrosis factor family, is a crucial receptor for apoptosis, and its ligand is a membrane protein located on the cell surface. Upon binding, Fas recruits Fas-associated protein with death domain (FADD) from the cytoplasm through its intracellular death domain. The death effector domain (DED) of FADD then interacts with Caspase-8 molecules (Tang et al., 2019). This interaction results in the formation of the death-inducing signaling complex (DISC), which consists of Fas, FADD, and Caspase-8. The presence of Caspase-8 within the DISC confers it with catalytic activity, thus activating the extrinsic apoptosis pathway in cells. In a previous investigation conducted by Shrestha et al., it was observed that the inhibition of FOXO3a gene activity hindered the amplification of FasL mRNA expression in breast cancer cells stimulated by globular adiponectin (gAcrp) (Ashkenazi et al., 2017; Yosefzon et al., 2018). These results imply that the signaling pathway involving FOXO3a could potentially have a crucial impact on the extrinsic apoptosis induced by fAcrp through the regulation of Fal gene expression (Amente et al., 2011).

**FIGURE 2:**
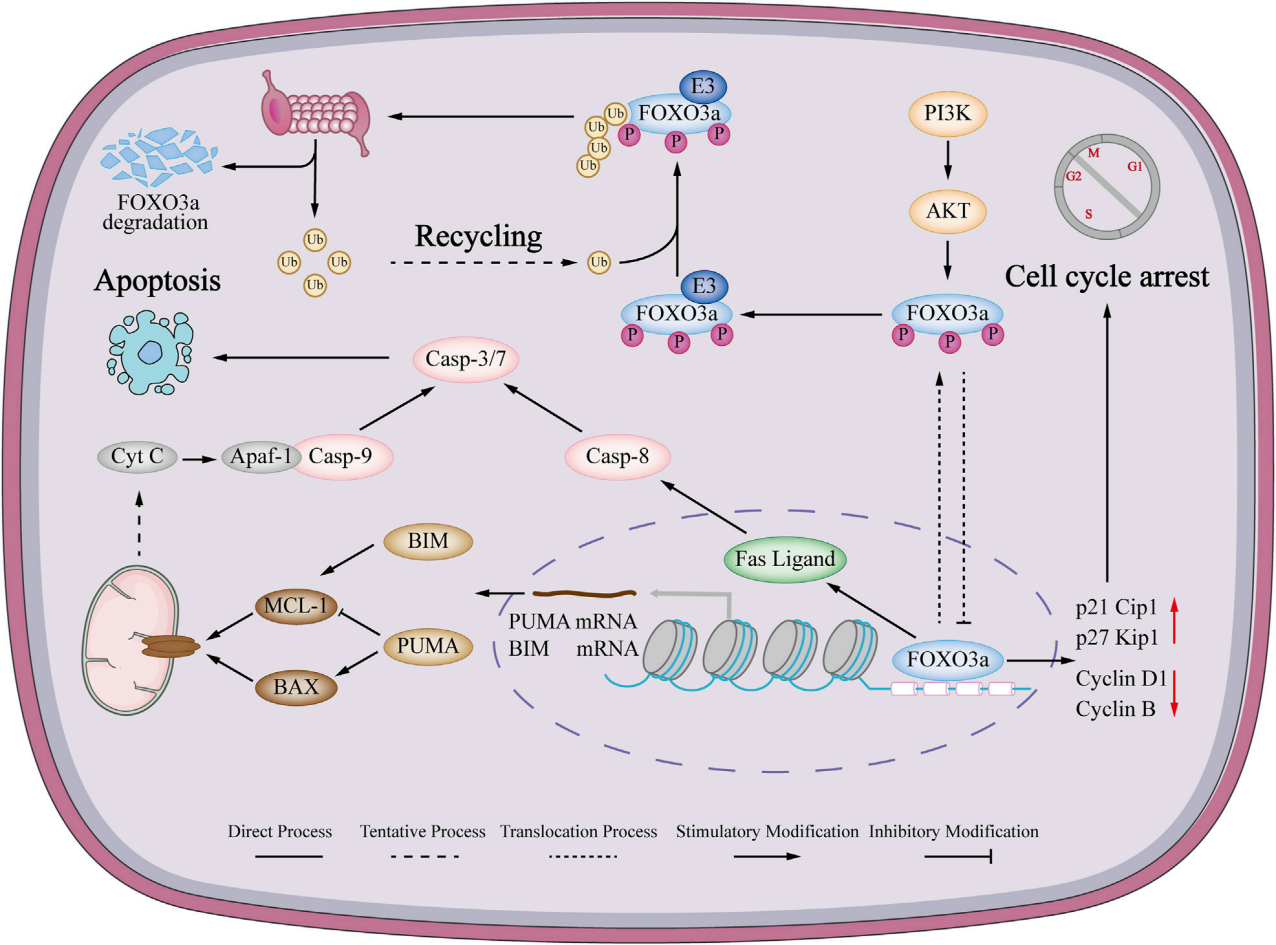
Mechanism of FOXO3a regulating apoptosis and cell cycle in breast cancer. FOXO3a can activate the endogenous mitochondrial signaling pathway in breast cancer cells, causing changes in mitochondrial membrane permeability, loss of transmembrane potential and release of cytochrome c. Immediately afterwards, cytochrome c forms a complex with Apaf-1 and Pro-cas-9, which ultimately promotes apoptosis. Also induces the extrinsic apoptotic pathway. FOXO3a can also promote the expression of FasL mRNA to induce cell extrinsic apoptosis. FOXO3a can promote the expression of p21Cip1 and p27kip1 and inhibit the expression of cyclinD1 and CyclinB to induce breast cancer cell cycle arrest.

Based on these previous investigations, it is indicated that FOXO3a is involved in both intrinsic and extrinsic apoptosis (Figure 2). An examination of the pharmacological impact of FOXO3a in breast cancer chemotherapy drugs, as well as its potential to argue the capacity of anticancer medications to trigger tumor cell apoptosis, will offer a novel approach to cure breast cancer.

### 5.2 FOXO3a and cell cycle in breast cancer

Many chemotherapeutic drugs and small molecule compounds inhibit cancer development by inducing cell cycle arrest. In the process of the cell cycle, phase-specific cyclins, cyclin-dependent kinases (CDKs), and Cip/Kip or INK family CDK inhibitors (CKIs) work together to regulate cell cycle through G1, S, G2, and M phases. Accumulating studies demonstrate FOXO3a is a significant candidate for cell cycle arrest in breast cancer (Roy et al., 2018; Wang et al., 2022) (Figure 2). Cellular communication network factor 5 (CCN5) can block the signaling pathway of phosphoinositide 3-kinase (PI3K)/AKT. It holds the capability to dephosphorylate the S318, S253 and Thr32 sites of FOXO3a, which consequently results in the nuclear relocation of FOXO3a from cytoplasm to nucleus and its subsequent activation. The activated FOXO3a, in a reciprocal manner, elevates the expression of p27Kp1 mRNA and obstructs the expression of Cyclin D1 (also known as CCND1) in breast cancer (Haque et al., 2015). In a separate study, it was found that when flotillin-1 (FLOT1) was silenced using RNAi in breast cancer, FOXO3a was activated. This activation led to an increase in the levels of p21Cip1 and p27kip1, which were cyclin-dependent kinase inhibitors, and a decrease in the expression of Cyclin D1 (Haque et al., 2015). In addition, small molecular compounds such as Flavone, apigenin and luteolin also induce cell cycle arrest in breast cancer cells, and the process also requires the participation of FOXO3a (Lin et al., 2015). These findings suggest that targeting FOXO3a could be a potential strategy to induce cell cycle arrest in breast cancer.

### 5.3 FOXO3a and EMT in breast cancer

**FIGURE 3:**
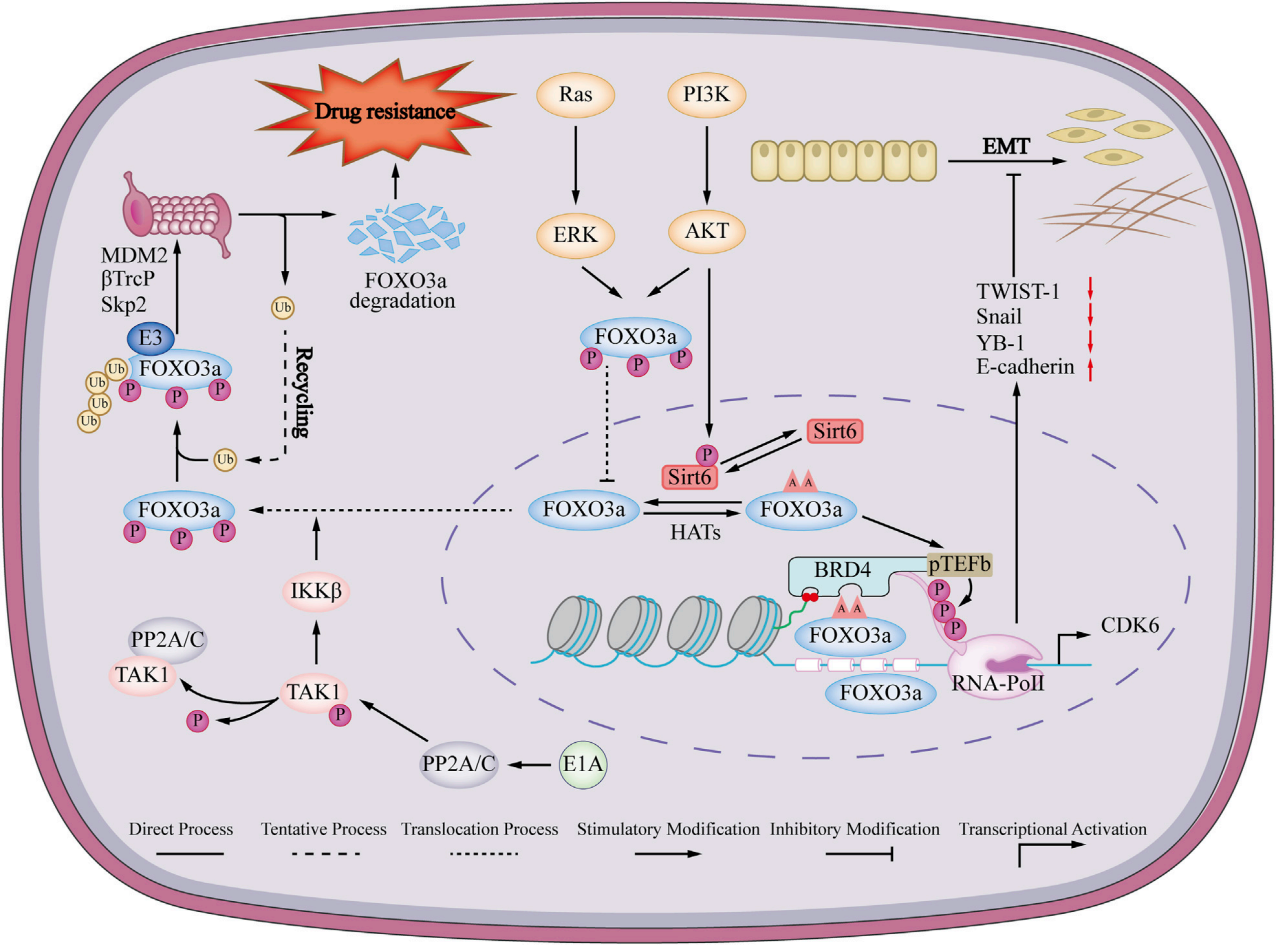
Mechanisms of FOXO3a regulating EMT and drug resistance in breast cancer. FOXO3a promotes the expression of epithelial marker E-cadherin and inhibits the expression of mesenchymal markers vimentin, YB-1, Snail and Twist, thereby promoting epithelial-mesenchymal transition (EMT) in breast cancer. Ras/PI3K signaling pathway induces phosphorylation of FOXO3a, phosphorylated FOXO3a cannot enter the nucleus, and is eventually degraded by the ubiquitin-proteasome pathway, thereby promoting drug resistance in breast cancer cells.

Breast cancer patients face a significant risk of mortality due to metastasis, which is the main culprit behind their untimely demise. The metastatic progression of tumors encompasses a series of intricate stages, while the process of EMT emerges as a pivotal regulatory factor (Liu et al., 2019; Huang et al., 2020). EMT triggers a transformative phenomenon within epithelial cells, leading to the loss of cell polarity and intercellular adhesive connections. Consequently, these cells undergo a quasi-mesenchymal metamorphosis, amplifying their abilities to migrate and invade surrounding tissues (Tang et al., 2021; Hu et al., 2022). EMT refers to a physiological process characterized by the downregulation of epithelial markers, including E-cadherin and zonula occludens-1 (ZO-1), alongside the upregulation of mesenchymal markers like vimentin and N-cadherin (Setlai et al., 2022). FOXO3a controls Twist to mediate the miR10b/CADM2/FAK/AKT axis to suppress EMT in breast cancer (Jin et al., 2021). Following OSU-53, a novel allosteric adenosine 5′-monophosphate-activated protein kinase (AMPK) activator, for treatment of breast cancer cells, FOXO3a directly binds to the E-cadherin promoter and activates its transcription. AMPK activation was linked to a reduction in AKT phosphorylation and an increase in FOXO3a expression. This activation also led to an increase in the epithelial marker E-cadherin, while reducing the presence of mesenchymal markers such as vimentin, YB-1, Snail, and Twist (Figure 3). In breast cancer cell lines treated with OSU-53 and shRNA knockdown of FOXO3a, the levels of these EMT markers were observed to be reversed. This finding provides confirmation that OSU-53 regulates the EMT of breast cancer cells by targeting AKT-murine double minute 2 (MDM2), thereby promoting the activation of FOXO3a, which further validating OSU-53’s anti-EMT activity was mediated by FOXO3a (Chou et al., 2014). Madureira et al. conducted a study that showed the activation of ERα gene transcription by FOXO3a in breast cancer cells. ERα signaling has been found to promote an epithelial phenotype by inhibiting the synthesis of Snail, which suppresses E-cadherin expression (Belguise et al., 2007). These studies strongly support that FOXO3a plays a crucial role in EMT of breast cancer. Investigating the regulatory role of FOXO3a in EMT can offer novel strategies for enhancing the survival outcomes and therapeutic options available for individuals diagnosed with breast cancer.

### 5.4 FOXO3a and drug resistance in breast cancer

The abnormal regulation of FOXO3a has been proven to participate in the drug resistance of cancer by targeting many oncogenic signaling pathways to induce apoptosis and enhance autophagy. Understanding the pathogenesis of FOXO3a in breast cancer will provide new insights into the treatment of breast cancer opinion (Yang et al., 2019; Liu et al., 2022b). FOXO3a is low-expressed in breast cancer drug-resistant cells (Song et al., 2018; Pellegrino et al., 2019). The underlying mechanism is that the breast cancer susceptibility gene 1 (BRCA1) promotes the ubiquitination and proteasomal degradation of FOXO3a mediated by MDM2 and S- phase kinase-associated protein 2 (SKp2) (Blanchard et al., 2015). Re-expressed FOXO3a restores sensitivity to TAM *in vitro* and *in vivo* (Pellegrino et al., 2019). The underlying mechanism may be that FOXO3a binds to the integrin α5 promoter region, promotes the expression of integrin α5 and restores its sensitivity to TAM (Ricci et al., 2022). The adenovirus early region 1A (E1A) inhibits the interaction between E3 ligase β-transducin repeats-containing protein (β-TrCP) and FOXO3a by suppressing the phosphorylation of FOXO3a serine 644 through IκB kinase (IIK). As a result, the degradation of FOXO3a through the ubiquitin-proteasome pathway is impeded, which ultimately increases the sensitivity of breast cancer cells to paclitaxel (Su et al., 2011). At the same time, overexpression of forkhead box K2 (FOXK2) and upregulation of FOXO3a can also achieve the same effect (Nestal de Moraes et al., 2018). Another study showed that AKT inhibitors induced dephosphorylation of FOXO3a, disrupted its binding with Sirtuin 6 (Sirt 6), and eventually led to acetylation of FOXO3a, and the acetylated FOXO3a would recognize the beta 2 defensin (BD2) domain of bromodomain-containing protein 4 (BRD4), which could recruit the BRD4/RNA polymerase II (RNAPII) complex to cell division protein kinase 6 (CDK6) initiation and induce its transcription to promote drug resistance of AKT inhibitor (Liu et al., 2018). Dysregulation of the protein phosphatase 3 catalytic subunit beta (PPP3CB)-FOXO3a-miRNA axis in heceptin-resistant human epithelial growth factor receptor-2 (HER2)-positive breast cancer disrupts this feedback mechanism, leading to the overproduction of insulin like growth factor 2 (IGF2) in heceptin-resistant breast cancer cells. TRAIL-inducing compound 10 (Tic 10) can reduce the expression of cell division protein kinase 4 (CDK4) by activating FOXO3a, thereby reversing the resistance of the breast cancer T47D/5-Fu cells to 5-fluorouracil *in vitro* and *in vivo* (Song et al., 2018). In summary, FOXO3a agonists or BDR4/FOXO3a inhibitors may become a new direction for the treatment of breast cancer drug resistance (Figure 3).

## 6 Regulation of FOXO3a in breast cancer

In the context of breast cancer, ncRNA plays a significant role in regulating the expression of FOXO3a, either directly or indirectly. The activation of FOXO3a has been found to effectively inhibit the occurrence and progression of breast cancer. Therefore, exploring the potential ncRNA to regulate FOXO3a has emerged as a promising strategy for breast cancer treatment. Additionally, numerous compounds have shown the ability to regulate FOXO3a, thereby exhibiting the suppression efficacy on breast cancer. These compounds include paclitaxel, flavones, vernodalin, epigallocatechin-3- gallate (EGCG), eugenol, and quercetin (Belguise et al., 2007; Lin et al., 2015), all of which exert their inhibitory effects on breast cancer through their impact on FOXO3a signal transduction. However, it is important to note that further research is necessary to fully understand and optimize the treatment of breast cancer using compounds that target the FOXO3a signaling pathway. Therefore, in this review, we aim to explore the role of ncRNAs in regulating FOXO3a and discuss the potential of related compounds in targeting the signaling pathways involving FOXO3a.

### 6.1 ncRNAs that regulate FOXO3a

Numerous biological processes in cancer are influenced by a wide array of ncRNAs (Anastasiadou et al., 2018). By identifying complementary target sites in the 3′UTR, miRNAs effectively suppress gene expression at the post-transcriptional level (Lee and Dutta, 2009). lncRNA can interact with chromatin regulatory factors to modify genes and regulate gene expression, promote or inhibit the translation of target mRNA; it can also compete with miRNA to bind to mRNA and be processed into miRNA (Zhang et al., 2014). There are also cirRNA and snoRNAs, which are related to various life activities. In breast cancer, understanding the relationship between ncRNA and FOXO3a can provide new clues for breast cancer research.

#### 6.1.1 miRNA

Several miRNAs have been found to decrease the expression of FOXO3a in cancer cells. For instance, the overexpression of miRNA-96-5p in gastric cancer has been shown to inhibit the expression of FOXO3a. Similar findings have also been observed in breast cancer. It is reported miR-155 promotes glycolysis and tricarboxylic acid (TCA) cycle mediated by the cMYC/pyruvate kinase M2 isoform (PKM2) axis by inhibiting FOXO3a, laying the foundation for the energy required for breast cancer development (Kim et al., 2018). A previous study showed that the inactivation of FOXO3a resulted in decreased expression of miR-128-3p and miR-30a-5p. This in turn, led to the overexpression of IGF2 and a decrease in the sensitivity of breast cancer cells (Luo et al., 2021). In breast cancer, the overexpression of miR-21 plays a role in the relocalization of FOXO3a protein from the nucleus to the cytoplasm through the PI3K/AKT axis. As a result, the reduced FOXO3a in the nucleus inhibits the expression of miR-34b/c, which ultimately promotes the growth of breast tumors (Liu et al., 2015). Similarly, FOXO3a can also activate miR-29b-2 and miR-338 to target vascular endothelial growth factor A (VEGF-A) and nucleotide-binding oligomerization domain 1 (NRP1), respectively, to inhibit the metastasis of breast cancer (Song et al., 2021).

#### 6.1.2 lncRNA

lncRNAs also regulate the biological function of FOXO3a. LINC01355 interacts with FOXO3a protein and prolongs the half-life of FOXO3a protein, leading to transcriptional repression of Cyclin D1 to induce breast cancer cell cycle arrest (Ai et al., 2019). Under hypoxic conditions, decreased recruitment of FOXO3a in the LINC00926 promoter region inhibits the transcription of LINC00926, thereby increasing the expression of phosphoglycerate kinase 1 (PGK1) to promote Warburg effect in breast cancer cells (Chu et al., 2021). LINCO1355 interacts with FOXO3a and stabilizes this protein, thereby inhibiting Cyclin D1 expression and inducing cell cycle arrest in the G0/G1 phase of breast cancer cells.

#### 6.1.3 circRNA

**FIGURE 4:**
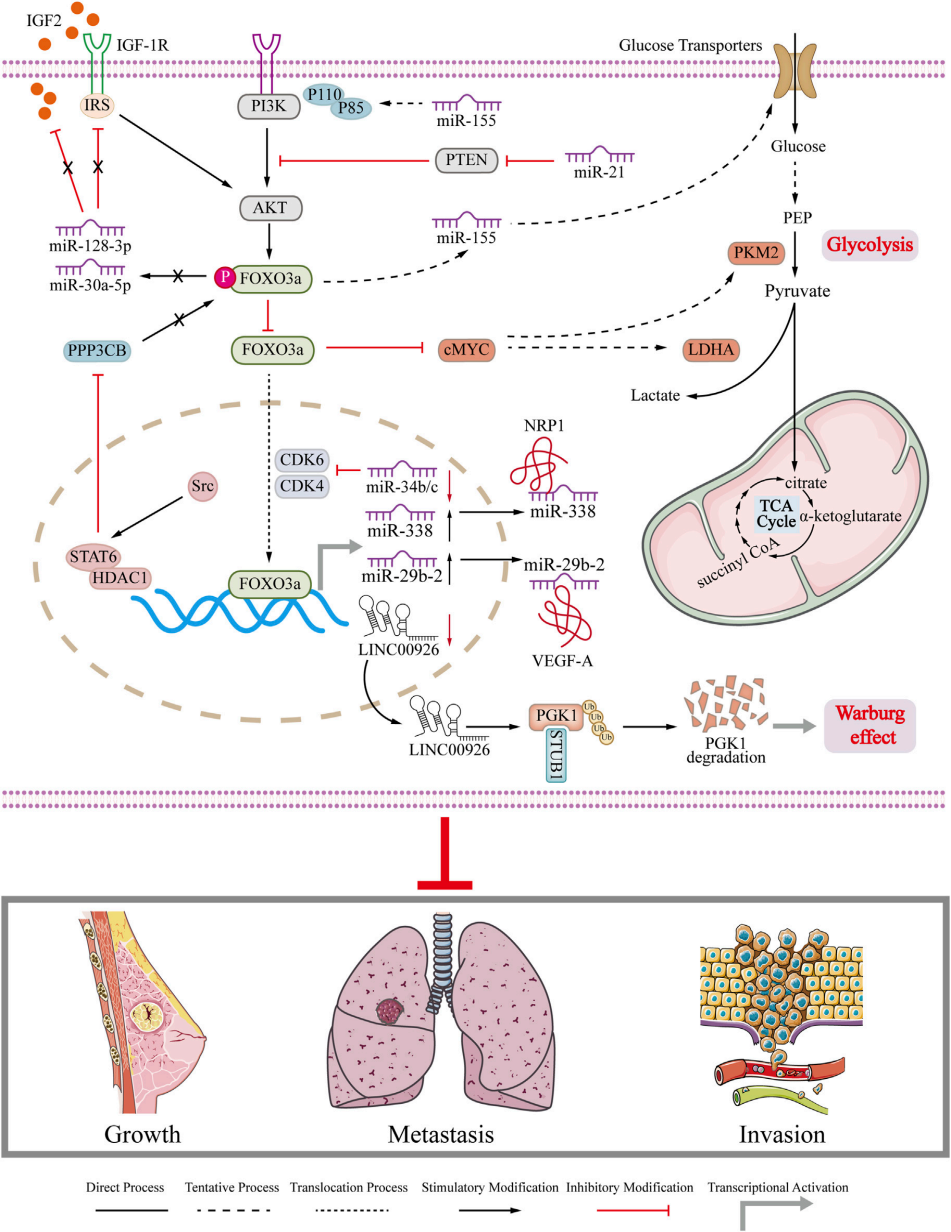
Regulation of FOXO3a in breast cancer. Schematic diagram illustrates that FOXO3a regulates related ncRNAs to regulate gene expression in breast cancer cells and the role of ncRNAs in breast cancer growth, metastasis and invasion.

The expression of circ-FOXO3a was found to be significantly decreased in breast cancer cell lines. In breast cancer cells with high levels of FOXO3a, the levels of p53 were reduced. This can be attributed to the role of circ-FOXO3a in promoting MDM2-induced degradation of p53 and simultaneously inhibiting MDM2-induced ubiquitination of FOXO3a (Du et al., 2017). Another report reveals Hsa_circ_0025202 competitively binds to miR-182-5p, relieves its inhibitory effect on FOXO3a expression, thereby inhibiting tumor progression and restoring the sensitivity of breast cancer cells to TAM (Sang et al., 2019). Moreover, some *in vivo* experiments prove that circRNA inhibits tumor growth and extends the lifespan of mice in breast cancer tumor animal model through activating FOXO3a. The role of FOXO3a interacted with ncRNA in breast cancer is summarized and depicted in Figure 4. The interaction between FOXO3a and ncRNA impairs glycolysis and Warburg effect in breast cancer and finally inhibits the growth, metastasis and invasion of breast cancer (Figure 4). The dysregulation of ncRNA in breast cancer plays a crucial role in the onset and progression of the disease. Therefore, gaining insight into the association between ncRNA and FOXO3a in breast cancer can improve the current treatment options for breast cancer. Summarized information is also presented in Table 1. Collectively, FOXO3a is involved in regulating apoptosis, cell cycle arrest, drug resistance, and EMT, and glycolysis in breast cancer (Liu et al., 2022b; Sun et al., 2023) (Figure 2; Figure 3; Figure 4). These biological behaviors are interconnected and not isolated. They are often initiated by the same signaling pathway, such as the PI3K/AKT pathway, which activates or inhibits FOXO3a, leading to its nuclear translocation and subsequent changes in downstream molecule expression. Additionally, phosphorylated FOXO3a undergoes degradation through the ubiquitin-proteasome system. These biological behaviors are closely associated with key processes like apoptosis and drug resistance in breast cancer cells. The authors propose that FOXO3a acts as a crucial link between different phenotypes observed in breast cancer cells. However, due to the limited studies in this area, further research is needed to explore the potential of targeting FOXO3a as a novel approach to breast cancer treatment.

**TABLE 1:** NcRNAs targeting FOXO3a in breast cancer.

ncRNA	Mechanism	Cell or animal model	References
miR-155	miR-155 promotes CMYC-mediated glycolysis by inhibiting FOXO3a	miR-155-deficient, breast cancer mouse model	Kim et al. (2018)
		Human primary breast cancer cell	
miR-29b-2/miR-338	FOXO3a activates miR-29b-2 and miR-338 to suppress breast cancer metastasis	MDA-MB-231, BT549	Song et al. (2021)
		Female nude mice	
miR-128-3p/miR-30a-5p	Dysregulation of the PPP3CB-FOXO3a-miRNA axis promotes drug resistance in HER2-positive breast cancer cells	SKBR3, BT474, pool2, HR20	Luo et al. (2021)
		Female BALB/c athymic nude mice	
miR-940	miR-940 promotes breast cancer progression by regulating FOXO3a	T47D, MCF-7	Zhang et al. (2020)
miR-21/miR-34b/c	miR-21 reduces FOXO3a protein level in the nucleus and inhibits miR-34b/c to promote breast tumor growth	MCF-10A, T47D, MDA-MB-231, MDA-MB-468	Liu et al. (2015b)
		BALB/c athymic nude mice	
miR-96	miR-96 downregulates FOXO3a to promote breast cancer cell proliferation	MCF-7, ZR-75–30	Lin et al. (2010)
miR-155-5p	Knockdown of miR-155-5p upregulates FOXO3a and promotes sensitivity of TNBC cells to bufalin	MDA-MB-231, MCF-7/ADR	Wang et al. (2016)
miR-10b	FOXO3a inhibits breast cancer EMT and metastasis through the TWIST-1/miR-10b/CADM2 axis	MCF-7, MDA-MB-231	Jin et al. (2021)
		Female nude mice	
miR-96-5p/miR-182-5p	miR-96-5p and miR-182-5p promote the occurrence and development of breast cancer by inhibiting FOXO3a	MCF-7, T47D	Yin et al. (2020), Mendes et al. (2023)
LINC01355	LINC01355 interacts with FOXO3a protein and prolongs FOXO3a half-life, leading to CCND1 transcriptional repression and induction of breast cancer cell cycle arrest	MCF-7, MDA-MB-231	Ai et al. (2019)
		Female BALB/c nude mice	
LINC00926	FOXO3a promotes LINC00926 transcription, leading to LINC00926 enhanced STUB1-mediated ubiquitination of PGK1, thereby suppressing glycolysis in breast cancer	MCF-7, MDA-MB-231 nude mice	Chu et al. (2021)
circ-FOXO3a	Circ-FOXO3a induces apoptosis of breast cancer cells by inhibiting MDM2-mediated ubiquitination of FOXO3a	BALB/c mice	Du et al. (2017)
		CD-1 nude mice	
		C57 mice	
circRNA_0025202	hsa_circ_0025202 acts as a sponge for miR-182-5p, thereby up-regulating FOXO3a downstream of miR-182-5p, thereby restoring the sensitivity of breast cancer to TAM.	MCF-7, T47D, MCF7/TR	Sang et al. (2019)
		Female BALB/c nude mice	

### 6.2 Compounds that regulate FOXO3a

#### 6.2.1 Paclitaxel

Drug resistance is a significant obstacle in breast cancer treatment, and substitution therapy has emerged as a key strategy to address this issue (Ali and Coombes, 2002; Zhang et al., 2022). Among them, taxane drugs are often used as classic drugs in breast cancer drug resistance, and their representative drugs include paclitaxel and its derivatives. They function by interacting with microtubules. However, the specific signal transduction pathway involved in it is still unclear. More and more studies have proved that FOXO3a plays an important role in the treatment of breast cancer with paclitaxel (Blagosklonny and Fojo, 1999; Symmans, 2001). Sunters et al. demonstrated that the nuclear translocation of FOXO3a increased in a dose-dependent manner in paclitaxel-treated MCF-7 cells (Nestal de Moraes et al., 2015). FOXO3a combined with the promoter of Bim and transactivated the expression of Bim, leading to the induction of intrinsic apoptosis. The study also revealed that silencing FOXO3a using siRNA significantly reduced paclitaxel-induced apoptosis in breast cancer cells. Further investigations indicated that paclitaxel-induced apoptosis in MCF-7 cells relied on the activation of c-Jun N-terminal kinase 1/2 (JNK 1/2) and the inhibition of AKT (Sunters et al., 2006). Inhibition of AKT in paclitaxel-treated cells facilitated the nuclear translocation of FOXO3a. Moreover, the addition of the JNK1/2 inhibitor SP600125 to paclitaxel-treated cells notably suppressed the nuclear translocation of FOXO3a and subsequently hindered the downstream intrinsic apoptosis of breast cancer cells. Based on these findings, it can be inferred that FOXO3a holds promise as a potential target for paclitaxel in the treatment of breast cancer (Sunters et al., 2003).

#### 6.2.2 Curcumin

Curcumin, a widely used phytochemical, has been extensively studied for its effectiveness and safety in preventing and treating various pathological conditions, including cancer (Li et al., 2019; Zhang et al., 2019). Notably, it has been found to increase the p21 level in MDA-MB-231 cells, regardless of the presence of p53 (Chiu and Su, 2009). These studies suggest that curcumin can be associated with both receptors and effectors of antiproliferative signaling in breast cancer (Cariou et al., 2000; Schmidt and Fan, 2001). It was discovered that the cytotoxic effects of curcumin to breast cancer cells may be influenced by the S-phase kinase-associated protein 2 (SKP2)-Cip/Kips pathway. Specifically, phosphorylation occurs continuously at certain sites of FOXO1 and FOXO3a in MCF-7 cells. However, curcumin works to prevent the dephosphorylation process of FOXO3a and FOXO1 in MDA-MB-231 cells. These findings suggest that MCF-7 cells utilize an alternative AKT survival pathway to avoid cell death by phosphorylating FOXO3a and FOXO1. Additionally, this explains why MDA-MB-231 cells are more susceptible to the effects of curcumin, while MCF-7 cells are resistant to curcumin (Jia et al., 2014).

#### 6.2.3 Vernodalin

Vernodalin, which is derived from the seeds of *Centratherum anthelminticum*, is a compound with cytotoxic properties. This sesquiterpene lactone contains two active α, β-unsaturated enoate moieties that display anti-tumor activity. When exposed to vernodalin, MCF-7 and MDA-MB-231 cells undergo apoptosis due to the activation of Bax, release of cytochrome C, and activation of caspases (Looi et al., 2013). Vernodalin dose-dependently induces an increase in the expression of FOXO3a in MCF-7 and MDA-MB-231 cells. The phosphorylation form of the key phosphorylation site (Ser253) of FOXO3a decreased in the second boom. However, siRNA silencing of FOXO3a could abolish the proliferation arrest and apoptosis after vernodalin treatment. In MCF-7 cells and MDA-MB231 cells, FOXO3a-specific siRNA inhibited the induction of p27Kip1, p21cip1/waf1 by vernodalin, and increased Cyclin D1 protein levels. *In vivo* immunohistochemistry (IHC) experimental data showed that vernodalin can induce tumor growth by inducing the expression of FOXO3a and p27Kip1 (Ananda Sadagopan et al., 2015).

#### 6.2.4 Epigallocatechin-3-gallate (EGCG)

Numerous epidemiological studies have consistently shown an inverse association between green tea consumption and the incidence of cancer. One of the key phenolic compounds in green tea is EGCG. In experiments using HER2/new-overexpressing breast cancer cells. Treatment with EGCG resulted in reduced activity of PI3K, AKT, and nuclear factor kappa B (NF-κB), as well as inhibited anchorage-independent growth. Not only that, EGCG promoted the exogenous expression of FOXO3a in rel-3875 and rel/CK2-5839 cells, and activated FOXO3a decreasing the level of Snail protein, a transcriptional repressor of the E-cadherin gene. Additionally, EGCG increased the expression of MTA3, E-cadherin, and g-catenin. In summary, the invasion of breast cancer was impeded through the activation of the FOXO3a/ERα/MTA3/E-cadherin pathway by EGCG (Belguise et al., 2007). Fulvia Farabegoli et al. also demonstrated that co-administration of EGCG and 6-OH-11-O-hydroxyphenanthrene (IIF) could promote FOXO3a and its nuclear import from the cytoplasm of MCF-7, MCF-7TAM and MDA-MB-231 cells to inhibit the growth of breast cancer (Farabegoli et al., 2014).

#### 6.2.5 Eugenol

Eugenol, a phenylpropanoid, is widely used in medicine as both a local anesthetic and antiseptic. Notably, it has been found to possess anticancer properties, particularly in breast cancer. Researchers have observed that eugenol can effectively inhibit the growth of both ER-negative and ER-positive breast cancer cells (Al-Sharif et al., 2013). Additionally, it could reduce the invasion and metastasis of MDA-MB-231 breast cancer cells by suppressing the expression of matrix metallopeptidase 2 (MMP-2) and matrix metallopeptidase 9 (MMP-9). Abdullah et al. found that the protein levels of AKT, FOXO3a, p21, p27, Caspase-3 and -9 were increased in eugenol-treated breast cancer cells. By targeting the FOXO3a/AKT signaling pathway, eugenol has the potential to enhance autophagy and apoptosis, ultimately inhibiting the growth and proliferation of breast cancer cells. However, further researches and studies are required to fully comprehend the therapeutic potential of eugenol and its effectiveness in breast cancer treatment (Abdullah et al., 2021).

#### 6.2.6 Quercetin

Quercetin, a flavonoid derived from plants, can be found in various fruits, vegetables, and teas. This compound exhibits numerous biological attributes including antioxidant, anti-inflammatory, and anticancer effects (Tang et al., 2020). Quercetin can induce intrinsic apoptosis and cell cycle arrest in TNBC cells. Nguyun et al. conducted a groundbreaking study that revealed the ability of quercetin to enhance the protein level of FOXO3a without altering its mRNA level. Moreover, they observed an increase in nuclear FOXO3a levels in breast cancer cells. Silencing endogenous FOXO3a expression protected breast cancer cells from apoptosis and cell cycle arrest, and abrogated the increased p53, p21, and growth arrest and DNA damage-inducible proteins (GADD45) signaling activity following quercetin treatment. Furthermore, the activation of FOXO3a by quercetin was antagonized by treatment with JNK inhibitor (SP600125), indicating that JNK might act as an upstream regulator of FOXO3a (Nguyen et al., 2017).

#### 6.2.7 Cardamonin (CD)

CD is an anticancer compound extracted from the seeds of the large black cardamom. Its potent anticancer properties have been extensively investigated in diverse cancer models (Nawaz et al., 2020). CD can induce apoptosis and cycle arrest in breast cancer cells, thus presenting a potential treatment for breast cancer. The underlying mechanism for the enhanced expression of FOXO3a and its target genes, such as p21, p27, and Bim, involves the activation of the upstream JNK. *In vivo* experiments demonstrated that CD inhibited the growth of MDA-MB-231 tumors. Consistent with the *in vitro* findings, the expressions of p-JNK, FOXO3a, p21, p27, and Bim were found to be upregulated in CD-treated tumor tissues. In summary, FOXO3a is involved in CD-induced cycle arrest and apoptosis in breast cancer cells (Kong et al., 2020).

#### 6.2.8 Gefitinib

Gefitinib is a specific inhibitor of epidermal growth factor receptor (EGFR). Krol et al. found that gefitinib can target the transcription factor FOXO3a and participate in the cell cycle arrest of BT474 and SKBR3 cells. In gefitinib-sensitive BT474 and SKBR3 cells, FOXO3a was dephosphorylated and translocated into the nucleus. In drug-resistant MDA-MB-453, MDA-MB-231 and MCF-7 cells, FOXO3a was retained in the cytoplasm (Krol et al., 2007). McGOvern et al. also found that gefitinib inhibited the expression of forkhead box protein M1 (FOXM1) in breast cancer cells through FOXO3a (McGovern et al., 2009). The findings propose that targeting FOXO3a offers novel avenue for enhancing the effectiveness of anti-EGFR medications and could also serve as valuable early indicators for forecasting treatment outcomes and monitoring anti-EGFR therapies.

#### 6.2.9 Withaferin A (WA)

WA is a bioactive compound derived from Withaferin (Agarwal et al., 1999). It exhibits various pharmacological effects, in many aspects such as regulating immune function, protecting the heart from ischemia-reperfusion injury, antibacterial, and anti-inflammatory. A previous study demonstrated that WA treatment significantly increased the protein levels of Bim isoforms in MDA-MB-231 and MCF cells. However, WA-mediated Bim was significantly abolished in breast cancer cells that silenced FOXO3a by siRNA. In conclusion, WA-induced intrinsic apoptosis of breast cancer cells depends on FOXO3a and Bim (Stan et al., 2008).

#### 6.2.10 Simvastatin

Statins are commonly used to lower blood lipids, and accumulating studies have proved that statins have anticancer activity (Duarte et al., 2021). Simvastatin was found to decrease phosphorylation of FOXO3a and increase total FOXO3a expression in SUM 159 and MDA-MB-231 cell lines, suggesting its potential impact on TNBC. Additionally, simvastatin was observed to induce cell cycle arrest and affect mammary gland formation and migration capacity. It is worth noting that the mRNA expression levels of FOXO3a in TNBC showed a positive correlation with metastasis-free survival. These findings suggest that simvastatin could play a curable role in inducing TNBC cell cycle arrest and inhibiting metastasis by regulating FOXO3a (Wolfe et al., 2015).

#### 6.2.11 Harmine hydrochloride (HMH)

HMH is a caroline alkaloid isolated from camel salsa seeds (Patel et al., 2012). It has been revealed signicant inhibitory effects on various malignant tumors such as gastric cancer, glioblastoma and hepatoblastoma (Liu et al., 2013; Zhang et al., 2014; Zhang et al., 2016; Tan et al., 2020). Ock et al. demonstrated that HMH could inactivate the PI3K/AKT/mammalian target of rapamycin (mTOR) signaling pathway in breast cancer cells, and upregulate the expression of FOXO3a, leading to cell cycle arrest in breast cancer (Ock and Kim, 2021). Furthermore, HMH treatment was observed to induce p38 phosphorylation in MCF-7 cells and JNK phosphorylation in MDA-MB-231 cells (Stan et al., 2008; Ock and Kim, 2021).

#### 6.2.12 5,7-Dihydroxy-8-nitrochrysin (NOC)

NOC is a synthetic chrysin analog that has been recently developed (Zhang et al., 2004). It has been found that NOC activates the mitochondrial apoptosis pathway in MDA-MB-231 cells. In these NOC-treated breast cancer cells, the expressions of Bim, Bax, p53, PUMA, and Noxa were upregulated, while the levels of Bcl-2 and Bcl-xl were decreased. When silencing FOXO3a using siRNA in breast cancer cells, NOC-induced apoptosis was significantly inhibited, and the expression of pro-apoptotic protein Bim decreased. In summary, NOC induces breast cancer cell apoptosis through the AKT/FOXO3a signaling pathway (Zhao et al., 2013).

#### 6.2.13 Fungal exocellular β-glucans

Fungal exocellular β-glucans have a direct cytotoxic effect on tumors and can also indirectly regulate immunity to suppress tumor growth. β-glucan BOTGLC, BOTFRU and LASGLC have been found to induce apoptosis and necrosis in MCF-7 breast cancer cells. The total expression of AMPK did not show significant differences among the three groups treated with β-glucans compared to the control group, but the phosphorylation of AMPK was significantly increased. Furthermore, treatment with BOTGLC and LAGLC for 48 h resulted in a significant increase in FOXO3a, while the phosphorylation of FOXO3a at Ser253 was significantly decreased. These findings suggest that both AMPK and FOXO3a are involved in β-glucans-induced apoptosis (Queiroz et al., 2015).

#### 6.2.14 Flavones

**FIGURE 5:**
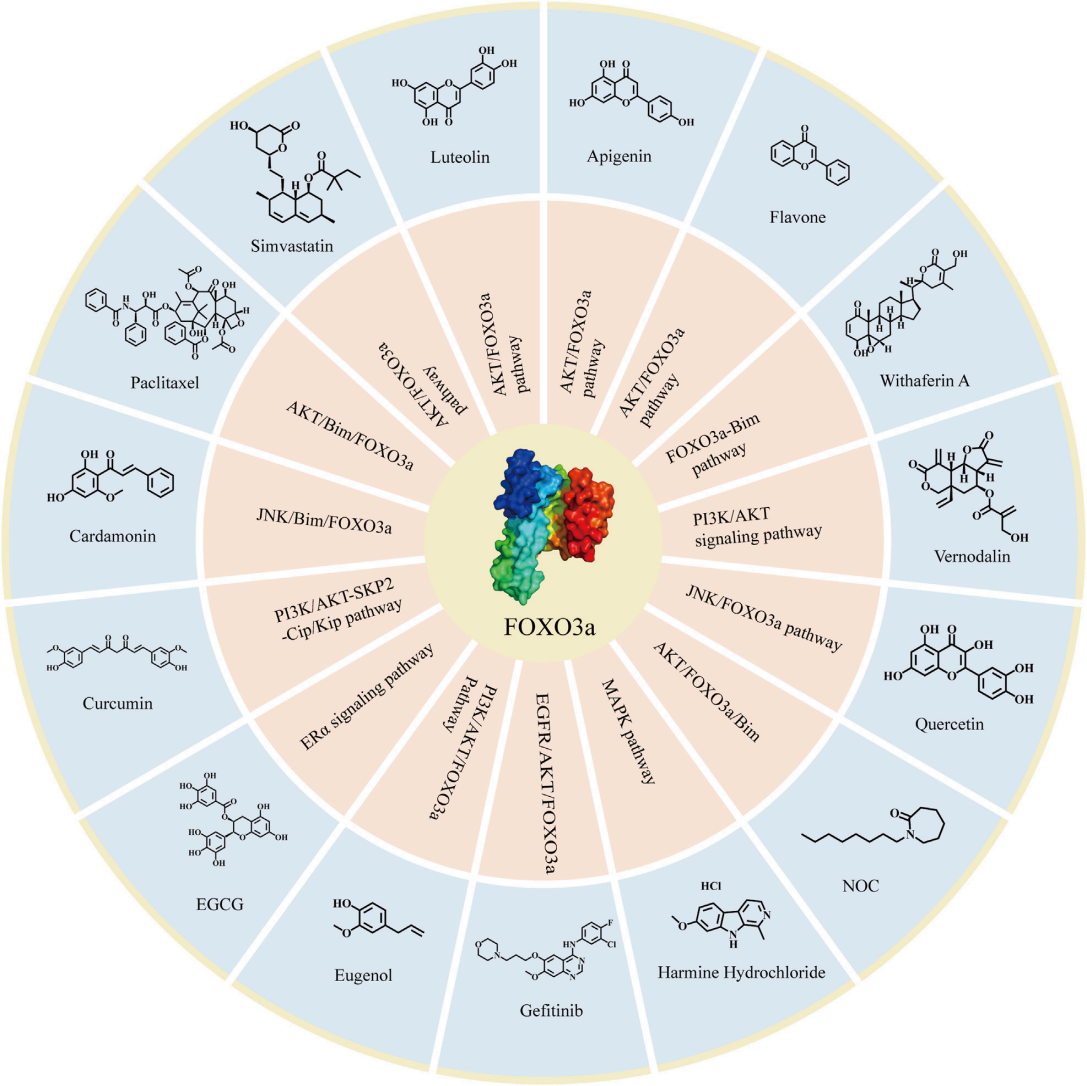
Compounds targeting FOXO3a in breast cancer. The compounds targeting FOXO3a and the related signaling pathways involved in attenuating breast cancer.

Flavone, apigenin, and luteolin are typical compounds belonging to the flavone subclass, known for their diverse beneficial biological properties including anti-inflammatory, antioxidant, and anti-tumor effects. In the study conducted by Lin et al., it was discovered that these flavones were able to induce breast cancer cell cycle arrest by enhancing the expression of p21 and p27 through the AKT/FOXO3a signaling pathway (Lin et al., 2015). Table 2 and Figure 5 summarize the detailed information about the above-mentioned compounds that target FOXO3a to inhibit breast cancer.

**TABLE 2:** The compounds targeting FOXO3a in breast cancer.

Compounds	Mechanism	Cell or animal model	References
Paclitaxel	Paclitaxel activates FOXO3a to induce breast cancer cell apoptosis	MCF-7, T47D, SKBR-7	Sunters et al. (2003), Sunters et al. (2006)
Flavones	Inhibits PIK/AKT activation and increases FOXO3a activation, thereby inducing breast cancer cell cycle arrest	MCF-7, Hs578T, MDA-MB -231	Lin et al. (2015)
Curcumin	AKT/SKP2 inhibitor combined with curcumin treatment enhances curcumin sensitivity in MDA-MB-231 cells and overcomes curcumin resistance in MCF-7 cells	MCF-7, MDA-MB -231	Jia et al. (2014)
Vernodalin	Vernodalin regulates the apoptosis of breast cancer cells and inhibits the growth of mammary tumors in rats by activating FOXO3a and its downstream targets	MCF-7, MDA-MB-231	Ananda Sadagopan et al. (2015)
		Pathogen-free female Sprague-Dawley rats	
EGCG	EGCG inhibits breast cancer invasion by activating FOXO3a/ERα/MTA3/E-cadherin signaling pathway	NF639, rel3875, rel/CK2-5839, MDA-MB-231	Belguise et al. (2007)
Eugenol	Eugenol induces autophagy and apoptosis in breast cancer cells by inhibiting PI3K/AKT/FOXO3a pathway	MDA-MB-231, SK-BR-3	Abdullah et al. (2021)
Quercetin	Quercetin induces apoptosis and cell cycle arrest in triple-negative breast cancer cells by activating FOXO3a	MD-MB-231	Nguyen et al. (2017)
Cardamonin	Cardamonin induces G2/M arrest and apoptosis in breast cancer cells by activating the JNK-FOXO3a pathway	MCF-10A, MDA-MB-231, MCF-7	Kong et al. (2020)
		Female nude mice	
Gefitinib	Gefitinib-induced FOXO3a dephosphorylation and nuclear translocation induces breast cancer cell cycle arrest and sensitivity to gefitinib	Bt474, SKBR3, MCF-7, MDA-MB-231, MDA-MB-453	Krol et al. (2007)
Withaferin A	WA induces breast cancer cell apoptosis through FOXO3a and Bim	MCF-7, MDA-MB-231, MCF-10A	Stan et al. (2008)
		Female nude mice	
Simvastatin	Simvastatin regulates FOXO3a to inhibit breast cancer metastasis	SUM 149, SUM159 Female SCID/Beige mice	Wolfe et al. (2015)
Harmine Hydrochloride	HMH induces breast cancer cell cycle arrest by regulating MAPK and AKT/FOXO3a signaling pathways	MCF-7, MDA-MB-231	Ock and Kim (2021)
NOC	NOC activates FOXO3a to induce breast cancer cell apoptosis	MDA-MB-453	Zhao et al. (2013)
β-glucans	Fungal exocellular β-glucans activate AMPK and FOXO3a to induce apoptosis in breast cancer cells	MCF-7	Queiroz et al. (2015)

Based on various interventions of the aforementioned compounds on FOXO3a and previous studies, it has been observed that these compounds exhibit promising clinical effects. Additionally, these compounds specifically target the FOXO3a pathway to inhibit breast cancer, and the inhibiting effectiveness of these compounds even significantly diminished when FOXO3a was eliminated (Manoharan et al., 2023). This shows that the inhibitory effects of these compounds on breast cancer must be mediated by FOXO3a. Therefore, there is ample reason to believe that FOXO3a is an emerging target for the treatment of breast cancer. Building upon this foundation, we have compiled the corresponding compound structures (Figure 5). This compilation will provide theoretical evidence and clinical application prospects for further research when treating FOXO3a as a target of functional proteins or structural proteins, and will facilitate the development of new drugs for breast cancer therapy.

## 7 Conclusion and perspective

Breast cancer is a kind of tumor with the highest incidence rate among female cancer patients (Haggerty and Purvis, 2021; Liu et al., 2022a). Although different therapies for different molecular subtypes of breast cancer have effectively improved the short-term prognosis of breast cancer patients, drug resistance still limits the effective survival of breast cancer patients (Liu et al., 2022b). Metastasis of breast cancer continues to be the leading cause of mortality among diagnosed patients. The growing evidence consistently highlights the significant contribution of FOXO3a in the progression and development of breast cancer. FOXO3a acts as a tumor suppressor in breast cancer, and its regulation is closely related to a variety of signaling pathways, and also involves the strict control of a complex network of ncRNAs and PTMs. The disorder of FOXO3a has been proven to be closely related to apoptosis, cell cycle arrest, EMT and drug resistance of breast tumors (Liu et al., 2018). Not only that, but it may also serve as a potential biomarker for breast cancer. The treatment methods for breast cancer patients are far from enough, and it is especially important to develop new methods for the treatment of breast cancer. FOXO3a is a potential therapeutic target in the treatment of breast cancer. Many molecular targeted drugs require the participation of FOXO3a, such as paclitaxel, simvastatin and so on. In addition, for the upstream and downstream signaling pathways of FOXO3a, many proteins that regulate the occurrence and development of tumors can mediate the occurrence and development of breast cancer by regulating FOXO3a. Identifying inhibitors of FOXO3a from nodes involved in the signaling pathway of FOXO3a may become a new strategy for the design of novel anticancer drugs for breast cancer. This current review recalls the FOXO3a′s related function and regulation in breast cancer and offers valuable insights for the development of innovative drugs that target FOXO3a. In conclusion, FOXO3a demonstrates potential as a drug target for breast cancer treatment, and the development of novel effective strategies based on FOXO3a may become a promising direction for breast cancer therapeutics.
